# Efficient enhancement of low-rank tensor completion via thin QR decomposition

**DOI:** 10.3389/fdata.2024.1382144

**Published:** 2024-07-02

**Authors:** Yan Wu, Yunzhi Jin

**Affiliations:** Yunnan Key Laboratory of Statistical Modeling and Data Analysis, Yunnan University, Kunming, China

**Keywords:** auxiliary variable tensor, tensor nuclear norm minimization, thin QR decomposition, Tucker decomposition, Tucker rank

## Abstract

Low-rank tensor completion (LRTC), which aims to complete missing entries from tensors with partially observed terms by utilizing the low-rank structure of tensors, has been widely used in various real-world issues. The core tensor nuclear norm minimization (CTNM) method based on Tucker decomposition is one of common LRTC methods. However, the CTNM methods based on Tucker decomposition often have a large computing cost due to the fact that the general factor matrix solving technique involves multiple singular value decompositions (SVDs) in each loop. To address this problem, this article enhances the method and proposes an effective CTNM method based on thin QR decomposition (CTNM-QR) with lower computing complexity. The proposed method extends the CTNM by introducing tensor versions of the auxiliary variables instead of matrices, while using the thin QR decomposition to solve the factor matrix rather than the SVD, which can save the computational complexity and improve the tensor completion accuracy. In addition, the CTNM-QR method's convergence and complexity are analyzed further. Numerous experiments in synthetic data, real color images, and brain MRI data at different missing rates demonstrate that the proposed method not only outperforms in terms of completion accuracy and visualization, but also conducts more efficiently than most state-of-the-art LRTC methods.

## 1 Introduction

With the rapid development of human needs and Internet technology, the scale of data acquired by people has expanded. Tensor data, such as third-order color images, fourth-order video sequences, and hyperspectral images, are now ubiquitous. At present, tensors are widely used in machine learning (Bai et al., 2021; Panagakis et al., 2021), computer vision (Kajo et al., 2019), image processing (Miao et al., 2020), etc., and are better at expressing the complex internal structure of higher-order data than vectors and matrices. However, due to data transmission and storage limitations, practical applications often face missing or corrupted observation tensor entries, which directly affect the quality of tensor analysis. Consequently, utilizing the observation part for missing tensor completion is a prominent research topic.

**Figure 1:**
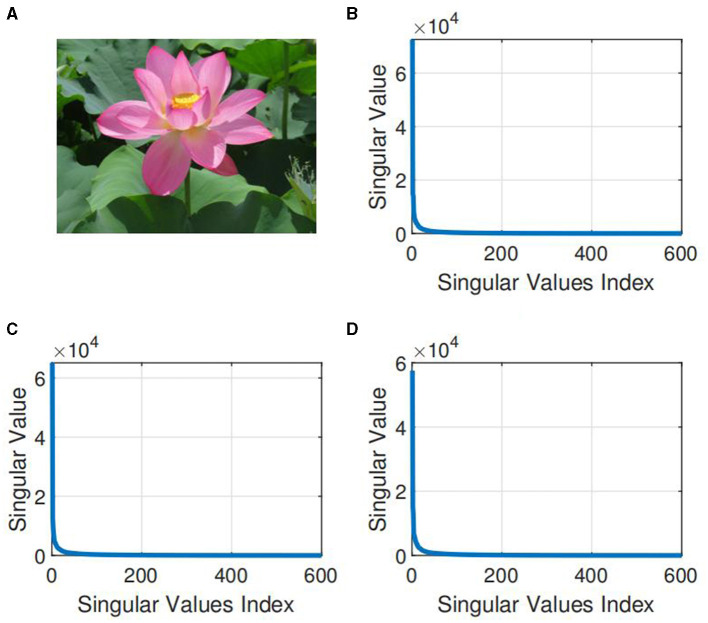
Illustration of the low-rank property of natural color image. **(A)** A three-dimensional low rank color image X with dimensions 800 × 600 × 3 (taken from https://www.pexels.com/jacquemay dominique). **(B)** Singular value distribution of the first channel X(:, :, 1). **(C)** Singular value distribution of the second channel X(:, :, 2). **(D)** Singular value distribution of the third channel X(:, :, 3).

Conventional methods of completion involve matrixization or vectorization of the tensor, which leads to dimensionality catastrophe in addition to destroying the spatial structure of high-order data. The aim of low-rank tensor completion (LRTC), a multidimensional expansion based on low-rank matrix completion (LRMC) (Candes and Recht, 2012; Xu et al., 2021), is to estimate missing terms (Zhou et al., 2018) by utilizing spatial correlation between tensor terms. LRTC is crucial for tensor processing and analysis. Several researches (Kolda and Bader, 2009; Liu et al., 2013; Zhao et al., 2020) have demonstrated that there is a significant quantity of redundant information in natural tensor data, which is typically low-rank or virtually low-rank, such as photos and videos. Figure 1 displays the distribution of singular values for the three channels of the color image. It can be observed that the majority information of the image is stored in only a few of the bigger singular values, its low rank structure is notable. This tensor property encourages the development of low-rank tensor estimation and completion problems, and it has been proactively applied in real-world problems like multi-channel image and video completion (Liu et al., 2013; Su et al., 2022), audio source separation (Yoshii et al., 2018), etc.

In the two-dimensional case, the rank is a strong tool for capturing global information about the data, but the definition of the tensor rank is not as explicit as that of the matrix, and the fundamental issue is that the definition of the tensor rank is not unique. The CANDECOMP/PARAFAC (CP) tensor rank is defined as the minimum number of rank-1 tensors needed in the CP decomposition. Nevertheless, CP rank determination is an NP-hard problem (Hillar and Lim, 2013), and there isn't always an optimal low CP rank approximation, which may result in a poor fit in real-world applications (Qiu et al., 2022). Another is the Tucker rank derived from the Tucker decomposition, defined as a vector composed of the ranks of mode unfolding matrices, which is extensively employed in LRTC driven by its superior compute-ability. In recent years, tensor train (TT) rank (Oseledets, 2011), tubal rank (Zhang and Aeron, 2016; Zhou et al., 2018), tensor ring (TR) rank (Yuan et al., 2019a), tensor tree rank (Liu et al., 2019b), etc. have also been successively proposed to provide an effective way to deal with the LRTC problem.

Owing to the nonconvex nature of the rank function, contemporary LRTC approaches predominantly employ the tensor nuclear norm as a convex substitute for the rank function. Liu et al. (2013) pioneered the definition of the tensor nuclear norm, modeled the LRTC as a convex optimization problem, and suggested three efficient algorithms to solve the LRTC problem. Shang et al. (2017) introduced a novel method for fuzzy dual nuclear norm minimization. In a related development, Bengua et al. (2017) leveraging the TT rank, proposed the SiLRTC-TT and TMac-TT models. These algorithms have significantly contributed to addressing the LRTC problem. Nevertheless, the majority of algorithms relying on tensor nuclear norm minimization (TNNM) encounter challenges associated with the elevated computational expense incurred by multiple SVDs. In addressing this issue, Liu et al. (2014a) formulates a novel tensor completion approach by imposing nuclear norm constraints on the factor matrices of CP decomposition. Shi et al. (2017) presented a tensor rank estimation approach based on *l*_1_-regularized orthogonal CP decomposition. Liu (2013) builds upon the TNNM, introduces the Tucker decomposition, and proposes a Core Tensor Nuclear Norm Minimization (CTNM) completion model.

While the CTNM model reduces the computational burden to a certain degree, the introduction of the Tucker decomposition inevitably involves the iterative solution of the factor matrix. A higher-order expansion of the matrix SVD to the tensor, the Higher Order SVD (HOSVD) (De Lathauwer et al., 2000a; Chao et al., 2021) was first proposed. De Lathauwer et al. (2000b) introduced the Higher-Order Orthogonal Iteration (HOOI) approach, which initializes the HOSVD solution, to boost approximation accuracy. These factor matrix solution techniques also involve multiple SVDs of tentor matricizations in the loop iterations, which surely places some computing strain on the CTNM model and contributes to its still-relatively-slow convergence rate. Building upon the insights from Shi et al. (2015), we adopt the thin QR decomposition, as opposed to HOOI, to address the factor matrix. We propose an efficient CTNM-QR model that circumvents the necessity for SVD in factor matrix resolution, thereby enhancing the algorithm's computational productivity. The principal contributions of this article are as follows:

(1) An enhanced CTNM-QR model is proposed, via introducing tensor versions of the auxiliary variables instead of matrices, while using the thin QR decomposition to solve the factor matrix instead of HOOI, which avoids the computation of multiple SVDs in each loop and minimizes the computational cost to a certain extent, thus speeding up the process.
(2) The complexity and convergence performance of the proposed method are analyzed. Compared with the state-of-the-art LRTC methods, the proposed method saves computational complexity, and is consistent with the generalized higher-order orthogonal iteration (gHOI) algorithm (Liu et al., 2014b) in terms of the algorithm's primary computational cost.
(3) The CTNM-QR model is applied to synthetic tensors, color images, and medical MRI scans for completion, validating the accuracy and efficacy of the enhanced algorithm.

The rest of the paper is organized as follows. We give the tensor notation and review some related work in Section 2. In Section 3, the proposed CTNM-QR method is described in detail, and the convergence and complexity of the CTNM-QR method are analyzed. In Section 4, we offer experimental analysis and completion results to validate the proposed algorithm. Finally, the paper is summarized in Section 5.

## 2 Notations and background

In this section, we introduce some notation and background of the tensor and tensor completion. Throughout this article, we use lowercase letters to denote vectors, e.g., *a, b*, uppercase letters for matrices, e.g., *A, B*, and uppercase fancy letters for tensors of order three and above, e.g., A, X. The *i*th element of a vector *a* is denoted as *a*_*i*_, the element of a matrix *A* with index (*i, j*) is denoted as *a*_*ij*_, and the elements of a *N*-order tensor $X\in {\mathbb{R}}^{I_{1}\times I_{2}\times \cdots I_{N}}$ are denoted as *x*_*i*_1_, *i*_2_, ⋯ , *i*_*N*__.

### 2.1 Tensor concepts and terminology

In this subsection, we first introduce some fundamental tensor concepts and terminology. An extensive review of tensors are discussed in Kolda and Bader (2009).

For the definition of a tensor, there are several definitions at different abstraction levels (Boumal, 2023). Here, an *N*th-order tensor is defined as a multidimensional array with *N* dimensions (*N* > 2), which is an extension of a matrix to higher order. Here, *N* represents the order of the tensor and the number of dimensions of the tensor, also known as modes. Analogous to the rows and columns of a matrix, we can create subarrays by fixing some of the given tensor's indices. A fiber of a tensor is a vector created by fixing all but one index of a particular mode (Filipović and Jukić, 2015). A third-order tensor X has row, column and tube fibers, denoted by *x*(:, *j, k*), *x*(*i*, :, *k*), *x*(*i, j*, :), respectively. A slice of a tensor is defined as a two-dimensional matrix, obtained by fixing all but the indices of two particular modes, e.g., a third-order tensor with horizontal, lateral, and frontal slices, denoted by *x*(*i*, :, :), *x*(:, *j*, :), and *x*(:, :, *k*), respectively.

We can also transform a tensor into a matrix via reordering the elements of an *N*th-order tensor, known as matricization, unfolding or flattening. The mode-*n* unfolding of an *N*th-order tensor $X\in {\mathbb{R}}^{I_{1}\times I_{2}\times \cdots \times I_{N}}$ is obtained by keeping the *n*th mode fixed and concatenating the slices of the rest of the modes into one long matrix, denoted by X_(*n*)_, which is an *I*_*n*_ × ∏_*j* ≠ *n*_*I*_*j*_ matrix for *n* = 1, 2, ⋯ , *N*, where the symbol ∏ is continued product. The tensor element (*i*_1_, *i*_2_, …, *i*_*N*_) is mapped into the element (*i*_*n*_, *j*) of the matrix X_(*n*)_, where


$$\begin{array}{l} j=1+\overset{N}{\underset{k=1,\neq n}{\sum }}\left(i_{k}-1\right)J_{k},J_{k}=\overset{k-1}{\underset{m=1,\neq n}{\prod }}I_{m}. \end{array}$$

The mode-*n* product of a tensor $X\in {\mathbb{R}}^{I_{1}\times I_{2}\times \cdots \times I_{N}}$ with a matrix $U\in {\mathbb{R}}^{J\times I_{n}}$ is denoted as $X\times_{n}U\in {\mathbb{R}}^{I_{1}\times \cdots \times I_{n-1}\times J\times I_{n+1}\times \cdots \times I_{N}}$. If there exists a series of distinct modes, the order of multiplication in the mode-*n* product is independent, that is,


$$\begin{array}{l} X\times_{m}A\times_{n}B=X\times_{n}B\times_{m}A\left(m\neq n\right). \end{array}$$

According to the mode-*n* unfolding of the tensor, we have the following formula


$$\begin{array}{l} Y=X\times_{n}U\Leftrightarrow Y_{\left(n\right)}=UX_{\left(n\right)}. \end{array}$$

The inner product of two tensors $X\in {\mathbb{R}}^{I_{1}\times I_{2}\times \cdots \times I_{N}}$ and $Y\in {\mathbb{R}}^{I_{1}\times I_{2}\times \cdots \times I_{N}}$ with the same size is denoted as a sum of the products of the corresponding elements, that is,


$$\begin{array}{l} \langle X,Y\rangle =\overset{I_{1}}{\underset{i_{1}=1}{\sum }}\overset{I_{2}}{\underset{i_{2}=1}{\sum }}\cdots \overset{I_{N}}{\underset{i_{N}=1}{\sum }}x_{i_{1}i_{2}\cdots i_{N}}y_{i_{1}i_{2}\cdots i_{N}}. \end{array}$$

By an extension of the matrix property, the Frobenius norm of the tensor $X\in {\mathbb{R}}^{I_{1}\times I_{2}\times \cdots \times I_{N}}$ is defined as the square root of the sum of the squares of all elements:


$$\begin{array}{l} \|X|{|}_{F}=\sqrt{\langle X,X\rangle }=\sqrt{\overset{I_{1}}{\underset{i_{1}=1}{\sum }}\overset{I_{2}}{\underset{i_{2}=1}{\sum }}\cdots \overset{I_{N}}{\underset{i_{N}=1}{\sum }}x_{i_{1}i_{2}\cdots i_{N}}^{2}}. \end{array}$$

The distance between the tensor X and Y is defined as ||X−Y||_*F*_.

The Kronecker product of two matrices *A* ∈ ℝ^*I*×*J*^ and *B* ∈ ℝ^*K*×*L*^ is denoted by *A* ⊗ *B*, and yields a matrix of size (*IK*) × (*JL*), defined as:


$$\begin{array}{l} A⊗B=\left[\begin{array}{cccc} a_{11}B & a_{12}B & \ldots & a_{1J}B\\ a_{21}B & a_{22}B & \ldots & a_{2J}B\\ \vdots & \vdots & \ddots & \vdots \\ a_{I1}B & a_{I2}B & \ldots & a_{IJ}B \end{array}\right]. \end{array}$$

### 2.2 Tensor decomposition

Tensor decomposition refers to expressing a tensor as a sequence of elementary operations on other simple arrays. There are many tensor decompositions. We introduce two major tensor decompositions, including CANDECOMP/PARAFAC (CP) decomposition (Cattell, 1944) and Tucker decomposition (Tucker, 1963).

For a given tensor $A\in {\mathbb{R}}^{I_{1}\times I_{2}\times \cdots \times I_{N}}$, the CP decomposition is defined as the factorization of A into the minimum number of linear combinations of rank-1 tensors:


$$\begin{array}{l} A=\overset{R}{\underset{r=1}{\sum }}\lambda_{r}{\text{a}}_{r}^{\left(1\right)}\circ \cdots \circ {\text{a}}_{r}^{\left(N\right)}, \end{array}$$

where “°” denotes the outer product of vectors, and ${\text{a}}_{r}^{\left(k\right)}\in {\mathbb{R}}^{I_{k}}\left(k=1,2,\;,R\right)$ is the factor vector.

The Tucker decomposition is based on an extension of the CP decomposition, a form of higher-order principal component analysis. The Tucker decomposition of the tensor A is expressed as:


$$\begin{array}{l} A\approx C\times_{1}U_{1}\times \cdots \times_{N}U_{N}, \end{array}$$

where $C\in {\mathbb{R}}^{R_{1}\times R_{2}\times \cdots \times R_{N}}$ is the core tensor and denotes the degree of interaction between the different components, and $U_{n}\in {\mathbb{R}}^{I_{n}\times R_{n}}$ is the *n*th-mode factor matrix, denoting the principal components on each mode, usually orthogonal. In general, *R*_*n*_ is much smaller than *I*_*n*_ for *n* = 1, ⋯ , *N*, so the core tensor C can be considered as a compressed version of the original tensor A, which reduces the computational complexity to some extent.

The matricized form of the Tucker decomposition is as follows:


$$\begin{array}{l} A_{\left(n\right)}=U_{n}C_{\left(n\right)}{\left(U_{N}⊗\ldots ⊗U_{n+1}⊗U_{n-1}⊗\ldots ⊗U_{1}\right)}^{\text{T}}. \end{array}$$

**Figure 2:**
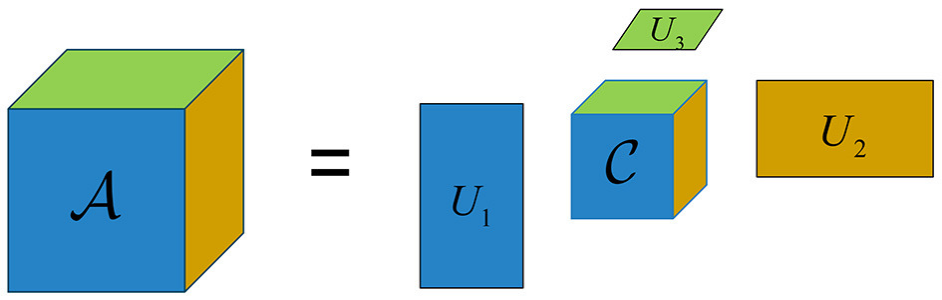
Illustration of the Tucker decomposition of a third-order tensor.

The Tucker-rank of the *N*th-order tensor $A\in {\mathbb{R}}^{I_{1}\times I_{2}\times \cdots \times I_{N}}$, also known as the multilinear rank (Kasai and Mishra, 2016), is denoted as an *N*-dimensional vector composed of the ranks of the various mode unfolding matrices of the tensor:


(1) $$\begin{array}{l} rank_{tc}\left(A\right)=\left(rank\left(A_{\left(1\right)}\right),rank\left(A_{\left(2\right)}\right),\;,rank\left(A_{\left(N\right)}\right)\right) \end{array}$$

where *rank*(A_(*n*)_) is the rank of the mode-*n* unfolding matrices, representing the dimension of the vector space spanned by the mode-*n* fiber. In contrast to the CP rank, the Tucker-rank can be easily obtained by computation, while the CP rank can only be determined empirically or by multiple experimental comparisons to search for the optimal value (Mu et al., 2014). Diagram of the Tucker decomposition of a third-order tensor is shown in Figure 2.

### 2.3 Low-rank tensor completion

The LRTC aims to infer and fill missing entries of the tensor from partially observed values via utilizing the low-rank structure of the tensor, which is an extension of the low-rank matrix completion.

Let *M* ∈ ℝ^*p*×*q*^ be the observed incomplete matrix and Ω be the index set, i.e., the observed positions of the matrix elements. Recovering the matrix *M* based on the observed partial elements is referred to as low-rank matrix completion, that is,


(2) $$\begin{array}{l} \underset{X}{\min}rank\left(X\right),\text{ s.t. }X_{\Omega }=M_{\Omega }, \end{array}$$

where the complete matrix *X* ∈ ℝ^*p*×*q*^ is obtained via the above optimization problem with constraints.

As a broadening of the low-rank matrix completion (Equation 2), the LRTC (Ji et al., 2009; Liu et al., 2013; Yuan et al., 2019a) is formulated as the following optimization problem:


$$\begin{array}{l} \underset{X}{\min}rank\left(X\right),\text{ s.t. }X_{\Omega }=T_{\Omega }, \end{array}$$

where $X,T\in {\mathbb{R}}^{I_{1}\times I_{2}\times \cdots \times I_{N}}$, T is the incomplete tensor containing the missing elements, and X is the completed tensor. The optimization problem is built with a rank minimization constraint, which ensures that the output tensor has the lowest rank possible for tensor completion. In the LRTC, there is also a category of optimization based on tensor decomposition, formulated as minimizing the discrepancy between the real observations and the predicted values to complete the missing entries under the given tensor rank. That is,


$$\begin{array}{l} \underset{X}{\min}:\|X_{\Omega }-T_{\Omega }{\|}_{F}^{2},\text{ s.t. }rank\left(X\right)=R, \end{array}$$

where *R* is a bounded constraint on the rank of the X and the tensor X fulfills a specific tensor decomposition model. The traditional models are the completion models based on the CP and Tucker decompositions, e.g., the optimization model for tensor completion using CP decomposition is stated as follows:


$$\begin{array}{l} \underset{X}{\min}\|X_{\Omega }-T_{\Omega }{\|}_{F}^{2},\text{ s.t. }X=\overset{R}{\underset{r=1}{\sum }}\lambda_{r}{\text{a}}_{r}^{\left(1\right)}\circ \cdots \circ {\text{a}}_{r}^{\left(N\right)}. \end{array}$$

The LRTC algorithm minimizing tensor rank is more widely used because the low-rank decomposition-based completion algorithm is impractical in some situations in reality, especially it is difficult to select an appropriate tensor rank for use in the algorithm when there are few observed elements. However, since the tensor rank is not unique, this makes the LRTC algorithm very challenging (Liu et al., 2013). Besides the CP rank and the Tucker rank in Equation (1), there are various forms of tensor rank, such as TT rank, tubal rank, TR rank, etc. Selecting one of these forms will result in different LRTC models.

### 2.4 Tensor nuclear norm minimization

In the LRTC, since the computation of the tensor CP rank is an NP-hard problem (Hillar and Lim, 2013) and the Tucker rank has the excellent property of being easy to compute, optimal completion models based on the Tucker rank of the tensor are widely used (Romera-Paredes and Pontil, 2013; Liu et al., 2019a; Yu et al., 2023). That is,


(3) $$\begin{array}{l} \underset{X}{\min}\overset{N}{\underset{i=1}{\sum }}\alpha_{i}rank\left(X_{\left(i\right)}\right),\text{ s.t. }X_{\Omega }=T_{\Omega }, \end{array}$$

where the parameters α_*i*_ are pre-specified weights and satisfy α_*i*_ ≥ 0, ∑α_*i*_ = 1. The Tucker rank of the tensor is represented in the form of a weighted sum. Gandy et al. (2011) suggests the unweighted model of α_*i*_ = 1(*i* = 1, 2, ⋯ , *N*).

Since the rank of the matrix is non-convex, it is difficult to guarantee to finding a globally optimal solution in the optimization (3). Since the nuclear norm of the matrix is shown to be the tightest convex envelop of the matrix rank (Candes and Recht, 2012), the completion model based on the nuclear norm minimization is proposed as follows:


(4) $$\begin{array}{l} \underset{X}{\min}\overset{N}{\underset{i=1}{\sum }}\alpha_{i}\|X_{\left(i\right)}|{|}_{*},\text{ s.t. }X_{\Omega }=T_{\Omega }, \end{array}$$

where the nuclear norm $\|X_{\left(i\right)}|{|}_{*}=\underset{j}{\sum }\sigma_{j}\left(X_{\left(i\right)}\right)$, and σ_*j*_(X_(*i*)_) denotes the *j*-th largest singular value of the matricization X_(*i*)_. The $\overset{N}{\underset{i=1}{\sum }}\alpha_{i}\|X_{\left(i\right)}|{|}_{*}$ is defined as the nuclear norm of the tensor X, denoted as ||X||_*_. The tensor nuclear norm is also known as the tensor schatten-q norm (Ji et al., 2009; Signoretto et al., 2010). Based on the nuclear norm, the concept of low-rank matrix completion is generalized to high-dimensional data, where the low-rank property of the tensor is often used as a necessary assumption to limit the degrees of freedom of missing entries (Kressner et al., 2014).

The tensor nuclear norm minimization completion model (TNNM) (4) can be expressed as the following:


(5) $$\begin{array}{l} \underset{X}{\min}\overset{N}{\underset{i=1}{\sum }}\alpha_{i}\|X_{\left(i\right)}|{|}_{*}+\frac{\lambda }{2}\|X_{\Omega }-T_{\Omega }{\|}_{F}^{2}, \end{array}$$

where λ > 0 is a regularization parameter.

Since the matrix nuclear norm in the model (5) is interdependent and cannot be optimized independently, auxiliary variables for splitting need to be introduced for simplifying solution algorithm. Liu et al. (2013) presented three solution algorithms, which are simple LRTC, fast LRTC, and high-accuracy LRTC. Based on tensor decomposition, Liu (2013); Liu et al. (2014a) proposed completion models for the factor matrix nuclear norm minimization and the core tensor nuclear norm minimization. Nevertheless, the majority of TNNM algorithms involve SVDs of several large matrices in each iteration, resulting in high computing costs.

## 3 The proposed method

### 3.1 Core tensor nuclear norm minimization based on thin QR decomposition

In the subsection, we describe the proposed CTNM-QR algorithm and the solution procedure in detail. Specifically, we adopt the ideas of the thin QR decomposition to update the factor matrix on the framework of the core tensor nuclear norm minimization (CTNM) and propose an efficient low-rank tensor completion algorithm.

In the CTNM model based on the Tucker decomposition (Liu, 2013; Liu et al., 2014b), the following assumption is stated:

Assumption 1. Let $X\in {\mathbb{R}}^{I_{1}\times I_{2}\times \ldots \times I_{N}}$ with *rank*_*tc*_ = (*R*_1_, *R*_2_, …, *R*_*N*_) and satisfy Tucker decomposition X = C ×_1_*U*_1_ ×_2_*U*_2_ × … ×_*N*_*U*_*N*_, where the core tensor $C\in {\mathbb{R}}^{R_{1}\times R_{2}\times \ldots \times R_{N}}$, and the orthogonal factor matrix $U_{i}\in {\mathbb{R}}^{I_{i}\times R_{i}}$. Then


$$\begin{array}{l} \|X|{|}_{*}=\|C|{|}_{*} \end{array}$$

where ||X||_*_ and ||C||_*_, denotes the nuclear norm of the original tensor and the core tensor, respectively.

According to the Assumption 1, the completion model for the CTNM is:


(6) $$\begin{array}{l} \begin{array}{c} \underset{C,\left\{U_{i}\right\},X}{\min}\overset{N}{\underset{i=1}{\sum }}\alpha_{i}\|C_{\left(i\right)}|{|}_{*}+\frac{\lambda }{2}\|X-C\times_{1}U_{1}\times \ldots \times_{N}U_{N}{\|}_{F}^{2},\\ s.t.,X_{\Omega }=T_{\Omega },\text{ and }{\left(U_{i}\right)}^{T}U_{i}=I_{I_{i}},i=1,2,\ldots ,N, \end{array} \end{array}$$

where *I*_*I*_*i*__ is an *I*_*i*_ × *I*_*i*_ Identity matrix. Owing to the interdependence of the unfolding matrices, we introduce auxiliary tensors $V_{i}\in {\mathbb{R}}^{R_{1}\times R_{2}\times \cdots \times R_{N}}\left(i=1,2,\;,N\right)$ to facilitate variable separation. Then, the model (6) is transformed into the following equivalent form:


(7) $$\begin{array}{l} \begin{array}{c} \underset{C,V_{i},U_{i},X}{\min}\overset{N}{\underset{i=1}{\sum }}\alpha_{i}\|V_{i\left(i\right)}|{|}_{*}+\frac{\lambda }{2}\|X-C\times_{1}U_{1}\times \cdots \times_{N}U_{N}{\|}_{F}^{2},\\ s.t.,X_{\Omega }=T_{\Omega },C=V_{i},\text{ and }U_{i}^{T}U_{i}=I_{I_{i}},i=1,\;,N. \end{array} \end{array}$$

For the optimization problem Equation (7), we propose an efficient CTNM-QR algorithm based on alternating direction multiplication method (ADMM) to address it. The ADMM is a commonly employed optimization method that decomposes a problem into a sequence of sub-problems to calculate the optimal solution, and is adept at proficiently addressing optimization problems featuring multiple non-smooth terms in the objective function (Glowinski, 2014; Boţ and Nguyen, 2020; Han, 2022). The partial augmented Lagrangian function for the model (7) is as follows:


(8) $$\begin{array}{l} \begin{array}{c} L\left(X,C,U_{1},\;,U_{N},V_{1},\;,V_{N},Y_{1},\;,Y_{N}\right)\\ =\overset{N}{\underset{i=1}{\sum }}\left(\alpha_{i}\|V_{i\left(i\right)}|{|}_{*}+\langle Y_{i},C-V_{i}\rangle +\frac{\mu }{2}\|C-V_{i}{\|}_{F}^{2}\right)\\ +\frac{\lambda }{2}\|X-C\times_{1}U_{1}\times \cdots \times_{N}U_{N}{\|}_{F}^{2}, \end{array} \end{array}$$

where Y_*i*_(*i* = 1, ⋯ , *N*) is the Lagrange multiplier tensor and μ > 0 is the penalty parameter.

The ADMM converts the original tensor completion problem (7) into solving the five subproblems of (9a–9e) by minimizing the augmented Lagrangian function (8), which iteratively updates the corresponding parameters. Specifically, we update $V_{i}^{k+1},C^{k+1},U_{i}^{k+1},X^{k+1}$ and $Y_{i}^{k+1}$ sequentially in the order of (9a–9e) with the fixed other parameters. The steps of the algorithm solution are as follows:


(9a) $$\begin{array}{l} V_{i}^{k+1}=\underset{V_{i}}{\arg\min}L\left(X^{k},C^{k},{\left\{U_{j}^{k},Y_{j}^{k}\right\}}_{j=1}^{N},{\left\{V_{j}^{k+1}\right\}}_{j=1}^{i-1},{\left\{V_{j}^{k}\right\}}_{j=i+1}^{N}\right), \end{array}$$

(9b) $$\begin{array}{l} C^{k+1}=\underset{C}{\arg\min}L\left(X^{k},C,{\left\{U_{j}^{k},V_{j}^{k+1},Y_{j}^{k}\right\}}_{j=1}^{N}\right), \end{array}$$

(9c) $$\begin{array}{l} U_{i}^{k+1}=\underset{U_{i}}{\arg\min}ℒ(X^{k},C^{k+1},{\left\{U_{j}^{k+1}\right\}}_{j=1}^{i-1},{\left\{U_{j}^{k}\right\}}_{j=i+1}^{N},\\ {\left\{V_{j}^{k+1},Y_{j}^{k}\right\}}_{j=1}^{N}),\text{s.t. }U_{i}^{T}U_{i}=I, \end{array}$$

(9d) $$\begin{array}{l} X^{k+1}=\underset{X}{\arg\min}L\left(X,C^{k+1},{\left\{U_{j}^{k+1},V_{j}^{k+1},Y_{j}^{k}\right\}}_{j=1}^{N}\right),\text{s.t.}X_{\Omega }=T_{\Omega }, \end{array}$$

(9e) $$\begin{array}{l} Y_{i}^{k+1}=Y_{i}^{k}+\mu^{k}\left(C^{k+1}-V_{i}^{k+1}\right). \end{array}$$

That is, for $V_{i}^{k+1}$ in the *k* + 1th iteration, (9a) can be written as the following optimization subproblem:


(10) $$\begin{array}{l} V_{i}^{k+1}=\underset{V_{i}}{\arg\min}\frac{\alpha_{i}}{\mu^{k}}\|V_{i\left(i\right)}|{|}_{*}+\frac{1}{2}\|C^{k}-V_{i}+\frac{Y_{i}^{k}}{\mu^{k}}{\|}_{F}^{2}\\ \text{for}i=1,2,\;,N. \end{array}$$

The iteration for $V_{i}^{k+1}$ for *i* = 1, 2, ⋯ , *N* can be effectively addressed through the application of the singular value thresholding (SVT) operator (Cai et al., 2010). It is easy to obtain a closed solution to the problem (10) as:


(11) $$\begin{array}{l} V_{i}^{k+1}={\mathrm{fold}}_{i}\left({\mathrm{SVT}}_{\alpha_{i}/\mu^{k}}{\left(C^{k}+\frac{Y_{i}^{k}}{\mu^{k}}\right)}_{\left(i\right)}\right), \end{array}$$

where fold_*i*_ denotes the transformation of the matrix into a tensor along the *i*th mode, if the SVD of a matrix *Z* is *Z* = *U*diag(σ)*V*^*T*^, ${\mathrm{SVT}}_{\tau }\left(Z\right)=U\mathrm{diag}\left(\max\left\{\sigma -\tau ,0\right\}\right)V^{T}$, max(·, ·) is the element-based maximization operator.

For C^*k*+1^ in the *k* + 1th iteration, the optimization problem (9b) with respect to C can be rewritten as:


(12) $$\begin{array}{l} C^{k+1}=\underset{C}{\arg\min}\overset{N}{\underset{i=1}{\sum }}\frac{\mu^{k}}{2}\|C-V_{i}^{k+1}+\frac{Y_{i}^{k}}{\mu^{k}}{\|}_{F}^{2}\\ +\frac{\lambda }{2}\|X^{k}-C\times_{1}U_{1}^{k}\times \cdots \times_{N}U_{N}^{k}{\|}_{F}^{2}. \end{array}$$

With other variables fixed, it is easy to see that Equation (12) is a smooth and differentiable optimization problem that can be derived by first-order optimality conditions for C (Liu, 2013; Liu et al., 2014b).

For X^*k*+1^ in the *k* + 1th iteration, solving X by the following subproblem:


(13) $$\begin{array}{l} X^{k+1}=\underset{X}{\arg\min}\|X-C^{k+1}\times_{1}U_{1}^{k+1}\times \cdots \times_{N}U_{N}^{k+1}{\|}_{F}^{2}\\ ,s.t.,X_{\Omega }=T_{\Omega }. \end{array}$$

By deriving the Karush-Kuhn-Tucker (KKT) condition for Equation (13), it is easy to obtain the update form of X as $X_{\Omega }^{k+1}=T_{\Omega }$ and $X_{\Omega^{c}}^{k+1}={\left(C^{k+1}\times_{1}U_{1}^{k+1}\times \cdots \times_{N}U_{N}^{k+1}\right)}_{\Omega^{c}}$, where Ω is the index set of the observed elements, Ω^*c*^ is the complement of Ω.

For the factor matrix $U_{i}^{k+1}\in {\mathbb{R}}^{I_{i}\times R_{i}}\left(i=1,2,\ldots ,N\right)$ in the *k* + 1th iteration, the optimization (9c) is equivalent to the following optimization problem:


(14) $$\begin{array}{l} U_{i}^{k+1}=\arg\min\|X^{k}-C^{k+1}\times_{1}U_{1}^{k+1}\cdots \times_{i-1}U_{i-1}^{k+1}\\ \times_{i+1}U_{i+1}^{k}\cdots \times_{N}U_{N}^{k}{\|}_{F}^{2}\text{ s.t. },{\left(U_{i}\right)}^{T}U_{i}=I_{I_{i}}, \end{array}$$

This is a peculiar Tucker decomposition model in which *U*_*i*_, *i* = 1, 2, …, *N* is an orthogonal matrix. HOSVD (De Lathauwer et al., 2000a; Chao et al., 2021) and HOOI (De Lathauwer et al., 2000b) are frequently used in the computation of Equation (14). Nevertheless, these approaches need alternating iterations of solving Equation (14) and involve SVDs of multiple tensor matricizations in each loop iteration, which causes the algorithms to converge quite slowly. To enhance the approaches, we adopt thin QR decomposition instead of HOOI for solving *U*_*i*_, which essentially avoid the SVDs during factorization and improve the computing efficiency.

Specifically, we first isolate the *i*th factor matrix *U*_*i*_ from the Tucker decomposition since the order of multiplications in the tensor mode-*i* product is not relevant. Then the equivalent form of the optimization model (14) for the solution of the *i*th factor matrix *U*_*i*_ is as follows:


(15) $$\begin{array}{l} \underset{U_{i}}{\min}\frac{1}{2}\|Z_{i}^{k}\times_{i}U_{i}-X^{k}{\|}_{F}^{2},\text{s.t.,}{\left(U_{i}\right)}^{T}U_{i}=I_{I_{i}}, \end{array}$$

where the given tensor $Z_{i}^{k}=C^{k+1}\times_{1}U_{1}^{k+1}\times_{2}\cdots \times_{i-1}U_{i-1}^{k+1}\times_{i+1}U_{i+1}^{k}\cdots \times_{N}U_{N}^{k}$. The problem (15) is nonconvex for all variables, yet it is convex for each block tensor. When the variables $C,X,{\left\{U_{j}\right\}}_{j=1,j\neq i}^{N}$ are fixed, the optimization (15) with respect to the factor matrix *U*_*n*_ is a smooth and differentiable optimization problem and can be solved via the first-order optimality condition. Hence the following equation holds:


$$\begin{array}{l} {\left(Z_{i}^{k}\times_{i}U_{i}-X^{k}\right)}_{\left(i\right)}Z_{i\left(i\right)}^{kT}=0, \end{array}$$

where ${\left(Z_{i}^{k}\times_{i}U_{i}-X^{k}\right)}_{\left(i\right)}$ and $Z_{i\left(i\right)}^{k}$ are the mode-*i* unfolding matrices of the tensors $Z_{i}^{k}\times_{i}U_{i}-X^{k}$ and $Z_{i}^{k}$, respectively. Then the least-squares solution without taking into account the orthogonality constraints on the factor matrices can be obtained by


$$\begin{array}{l} {\tilde{U}}_{i}^{k+1}=X_{\left(i\right)}^{k}Z_{i\left(i\right)}^{kT}{\left(Z_{i\left(i\right)}^{k}Z_{i\left(i\right)}^{kT}\right)}^{-1}. \end{array}$$

Motivated by the work of Shi et al. (2015), we adopt the thin QR decomposition for the orthogonality constraints of the factor matrices, that is,


(16) $$\begin{array}{l} U_{i}^{k+1}=QR\left({\tilde{U}}_{i}^{k+1}\right),\text{for}i=1,2,\ldots ,N, \end{array}$$

where *QR*(·) denotes the thin *QR* decomposition of the matrix. At this step, the proposed CTNM-QR algorithm merely needs to carry out QR decomposition of a matrix with the computational complexity $O\left(\overset{N}{\underset{i=1}{\sum }}I_{i}\times R_{i}^{2}\right)$. While the computational complexity of the HOSVD algorithm in terms of *U*_*i*_ iterations is $O\left(\overset{N}{\underset{i=1}{\sum }}I_{i}^{2}\times \Pi_{j\neq i}I_{j}\right)$, the HOOI algorithm is $O\left(\overset{N}{\underset{i=1}{\sum }}I_{i}^{2}\times \Pi_{j\neq i}R_{j}\right)$, where *R*_*i*_ ≪ *I*_*i*_. This indicates that the significant computational complexity associated with the SVD can be effectively mitigated by employing QR decomposition to resolve the factor matrix.

### 3.2 Implementation

In the following, we discuss several implementations issues. To effectively solve the objective function (8), the penalty parameter μ is adaptively varied via setting the initialization of μ to μ_0_, and iteratively increasing μ^*k*^ by μ^*k*+1^ = ρμ^*k*^, where ρ ∈ (1.0, 1.1] and μ_0_ is usually a smaller constant. For the iteration among the problems (9a–9e), we principally use two convergence criteria. One is the relative change satisfaction between two neighboring recovery tensors X^*k*+1^ and X^*k*^:


$$\begin{array}{l} \frac{\|X^{k+1}-X^{k}|{|}_{F}}{\|X^{k}|{|}_{F}}\leq tol, \end{array}$$

where *k* is the number of iterations and *tol* is the tolerance error. The alternative halting condition is *k* ≥ *K*, where *K* is the maximum number of iterations. The iteration ends if any of the two conditions is met. We summarize the implementation of the CTNM-QR as Algorithm 1.

**Algorithm 1:**
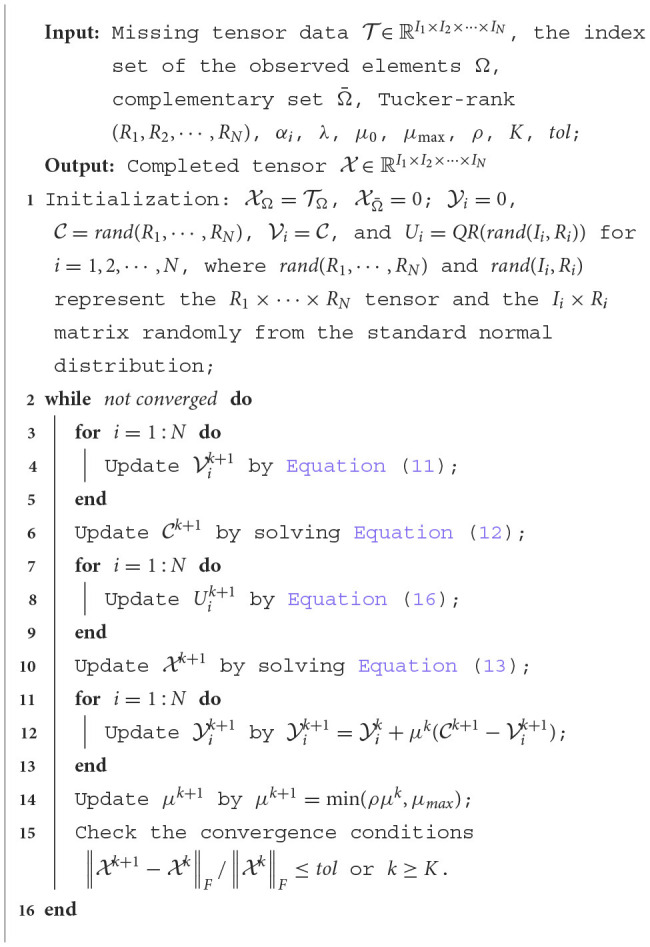
Core tensor nuclear-norm minimization with QR decomposition (CTNM-QR).

### 3.3 Convergence analysis

Let $\left\{C^{k},U_{1}^{k},\ldots ,U_{N}^{k},V_{1}^{k},\ldots ,V_{N}^{k},X^{k}\right\}$ be a sequence generated by the Algorithm 1. Although the CTNM-QR is a nonconvex ADMM algorithm, the subsequence are convergent and converge to the KKT point of the problem (6) proved by Liu et al. (2014b). In the subsection, we only illustrate the convergence of the improved part of the algorithm for the subproblem (9c).

Denote the objective function in Equation (15) as $f\left(U_{n}\right)=\frac{1}{2}\|Z_{n}\times_{n}U_{n}-X{\|}_{F}^{2}$, where ${\left(U_{n}\right)}^{T}U_{n}=I_{I_{n}}\left(n=1,2,\ldots ,N\right)$. The function *f*(*U*_*n*_) is convex on *U*_*n*_ without considering orthogonality constraints, so minimizing *f*(*U*_*n*_) yields ${\tilde{U}}_{n}^{k}=X_{\left(n\right)}^{k-1}{\left(Z_{n\left(n\right)}^{k-1}\right)}^{T}{\left(Z_{n\left(n\right)}^{k-1}{\left(Z_{n\left(n\right)}^{k-1}\right)}^{T}\right)}^{-1}$. Then the following equation holds:


$$\begin{array}{l} f\left({\tilde{U}}_{n}^{k}\right)\leq f\left(U_{n}^{k-1}\right). \end{array}$$

Denote $g\left(C^{k},U_{n}\right)=\frac{1}{2}\|C^{k}\times_{1}U_{1}^{k}\cdots \times_{n-1}U_{n-1}^{k}\times_{n+1}U_{n+1}^{k-1}\cdots \times_{N}U_{N}^{k-1}\times_{n}{\tilde{U}}_{n}-X^{k}{\|}_{F}^{2}$. By carrying out a thin QR decomposition of ${\tilde{U}}_{n}^{k}$, we have ${\tilde{U}}_{n}^{k}=U_{n}^{k}Q^{k}$, where $U_{n}^{k}$ is an orthogonal matrix. Then we have


$$\begin{array}{l} f\left({\tilde{U}}_{n}^{k}\right)=g\left(C^{k},{\tilde{U}}_{n}^{k}\right)=g\left({\tilde{C}}^{k},U_{n}^{k}\right)=f\left(U_{n}^{k}\right)\leq f\left(U_{n}^{k-1}\right) \end{array}$$

where ${\tilde{C}}^{k}=C^{k}\times_{n}Q^{k}$. Therefore, when $C,X,{\left\{U_{i}\right\}}_{i=1,i\neq n}^{N}$ is fixed, the objective function *f*(*U*_*n*_) tends to be decreasing during the iteration process, and the sequence $\left\{U_{1}^{k},\ldots ,U_{N}^{k}\right\}$ is the convergence sequence.

### 3.4 Complexity analysis

In the subsection, we analyze the complexity of the proposed CTNM-QR algorithm. The running time of the CTNM-QR algorithm is primarily consumed in solving the auxiliary variables with the SVT operator and some multiplicative computations. Suppose the size of the input tensor is *I*_1_ × *I*_2_ × ⋯ × *I*_*N*_ and Tucker rank is *R*_1_ = ⋯ = *R*_*N*_ = *R*, the time complexity of solving Equation (9a) is O(*NR*^*N*+1^), the time complexity of solving X in (9d) is $O\left(R\prod_{i=1}^{N}I_{i}\right)$, and the time complexity of computing *U*_*n*_ in Equation (16) is $O\left(NR\prod_{i=1}^{N}I_{i}+R^{2}\underset{i}{\sum }I_{i}\right)$. Consequently, the total time complexity of the Algorithm 1 is $O\left(T\left(N+1\right)R\prod_{i=1}^{N}I_{i}\right)$, where *T* is the total number of iterations. When the *N* and *I*_*i*_, *i* = 1, 2, ⋯ , *N* are fixed, the time complexity of the CTNM-QR algorithm mainly dominates on *R*. Fortunately, the *R* generally takes small values due to the low-rank assumption of the tensor.

For comparison, we list the major computational complexity of several classical tensor-completion algorithms in Table 1. Although the per-iteration complexity of TT-SGD (Yuan et al., 2019b) is incredibly low, the total number of iterations required is higher and more time-consuming than the proposed CTNM-QR algorithm in practice. In addition, the Polak-Ribiere nonlinear conjugate gradient algorithm of CP-WOPT (Acar et al., 2011) also suffers from the problem of being time-consuming. Compared with the nuclear norm minimization algorithms such as SiLRTC, HaLRTC, CTNM, etc., the proposed CTNM-QR algorithm saves some computational complexity. The CTNM-QR is consistent with the generalized higher-order orthogonal iteration (gHOI) algorithm (Liu et al., 2014b) in terms of the algorithm's primary computational cost.

**Table 1:** Computational complexity of each iteration in tensor completion algorithms.

**Algorithms**	**Complexity**
TT-WOPT (Yuan et al., 2017)	$O\left(N\prod_{i=1}^{N}I_{i}+R^{2}\overset{N}{\underset{i=1}{\sum }}\prod_{j\neq i}I_{j}\right)$
TT-SGD (Yuan et al., 2019b)	O(*N*^2^*R*^3^)
CP-WOPT (Acar et al., 2011)	$O\left(2\left(N+1\right)R\prod_{i=1}^{N}I_{i}\right)$
SiLRTC, HaLRTC (Liu et al., 2013)	$O\left(\overset{N}{\underset{i=1}{\sum }}I_{i}\prod_{i=1}^{N}I_{i}\right)$
CTNM (Liu, 2013)	$O\left(\left(2N+1\right)R\prod_{i=1}^{N}I_{i}\right)$
gHOI (Liu et al., 2014b)	$O\left(\left(N+1\right)R\prod_{i=1}^{N}I_{i}\right)$
CTNM-QR	$O\left(\left(N+1\right)R\prod_{i=1}^{N}I_{i}\right)$

## 4 Experiments

In this section, we conduct experiments to evaluate the performance and efficiency of the CTNM-QR algorithm in the LRTC problem using synthetic tensor data, color images, and medical MRI scans. The experiment compares the CTNM-QR with five other typical completion algorithms: gHOI (Liu et al., 2014b), CTNM (Liu, 2013), FaLRTC (Liu et al., 2013), CP-WOPT (Acar et al., 2011), and TT-SGD (Yuan et al., 2019b), where the parameters of the comparison algorithms are optimized. In addition, by means of studies, we analyze the convergence of the CTNM-QR algorithm intuitively. All the experiments are implemented on Windows 10 and MATLAB (R2018b) with an Intel(R) Core(TM) i5-7200U CPU at 2.70 GHz and 4 GB RAM.

To construct the incomplete tensor artificially, we utilize random uniform sampling from the complete tensor based on the given missing rate (MR), so the tensor missing form is randomized uniform missing. We set up varying MR for the test tensor, where the MR is defined as:


$$\begin{array}{l} MR=1-\frac{M}{\prod_{n=1}^{N}I_{n}} \end{array}$$

where *M* is the number of entries observed in the *N* order tensor $T\in {\mathbb{R}}^{I_{1}\times I_{2}\times \cdots \times I_{N}}$. We adopt relative squared error (RSE) and CPU time as evaluation metrics to measure the performance of the tensor completion. The RSE is calculated as follows:


$$\begin{array}{l} RSE=\frac{\|X-T|{|}_{F}}{\|T|{|}_{F}} \end{array}$$

where ||·||_*F*_ indicates the Frobenius norm, the X and T denote the tensor recovered by the algorithm and the original true tensor, respectively. The algorithm's accuracy in recovering the tensor increases as the RSE decreases, and computational complexity decreases as the CPU time decreases.

### 4.1 Synthetic tensor completion

In the simulation studies, we generate the low rank synthetic tensor $T\in {\mathbb{R}}^{I_{1}\times I_{2}\times \cdots \times I_{N}}$ by the Tucker decomposition model, i.e., T = C ×_1_*U*_1_ ×_2_*U*_2_ × … ×_*N*_*U*_*N*_, where $C\in {\mathbb{R}}^{R_{1}\times R_{2}\times \ldots \times R_{N}}$, $U_{i}\in {\mathbb{R}}^{I_{i}\times R_{i}}\left(i=1,2,\ldots ,N\right)$ and their entries independently follow the standard normal distribution. This implies that the Tucker rank of the tensor T is (*R*_1_, *R*_2_, …, *R*_*N*_).

In the concrete implementation, we set the tolerance to *tol* = 10^−5^ for all algorithms, the maximum number of iterations to *K* = 6 × 10^5^ for the TT-SGD, and *K* = 500 for the rest of the algorithms. For the FaLRTC, gHOI, CTNM, and CTNM-QR, we set the weights α_*i*_ = 1/*N, i* = 1, 2, ⋯ , *N*. The smoothing parameter of the FaLRTC is set to α_*i*_/*I*_*i*_, *i* = 1, …, *N*, and the rest of the parameters are maintained at their default values. For the CP-WOPT, the tensor rank *R* is set to 15. For the TT-SGD, the TT rank is set to *R*_1_ = ⋯ = *R*_*N*−1_ = 15. For the gHOI, CTNM, and CTNM-QR, we set $\mu_{0}=10^{-3}$, $\mu_{\max}=10^{10}$, ρ = 1.05.

We consider two cases including the fourth-order tensor with the size 40 × 40 × 40 × 40 and the fifth-order tensor with the size 20 × 20 × 20 × 20 × 20. The Tucker-rank of the tensor is set to *R*_1_ = ⋯ = *R*_*N*_ = 5, where the tensor orders *N* are 4 and 5, respectively. We consider four distinct MRs 30, 50, 70, 80% for the test tensor and replicate 100 simulations. Table 2 presents the average results (RSE and time cost) for 100 independent experiments. The outcomes reveal that the CTNM-QR method yields more accurate solutions with the less time costs, and outperforms other algorithms in the term of the RSE regardless of whether it's a higher order tensor or a lower order tensor. Although the gHOT method has a somewhat shorter running time than the CTNM-QR at the low MRs, the time costs of both methods are quite close and the RSE of the CTNM-QR is less than one of the gHOI.

**Table 2:** Average RSE and time cost (seconds) of all approaches on various tensor sizes.

	**CP-WOPT**		**FaLRTC**		**TT-SGD**		**CTNM**		**gHOI**		**CTNM-QR**	
**MR**	**RSE**	**Time**	**RSE**	**Time**	**RSE**	**Time**	**RSE**	**Time**	**RSE**	**Time**	**RSE**	**Time**
40 × 40 × 40 × 40												
30%	4.62e-1	73.96	1.47e-2	44.12	5.99e-2	138.25	2.40e-3	9.75	6.73e-4	**2.75**	**3.71e-4**	3.06
50%	5.54e-1	100.21	2.95e-2	38.67	9.77e-2	136.57	8.96e-3	20.15	2.36e-3	**4.97**	**1.91e-3**	5.56
70%	5.73e-1	144.19	5.61e-2	63.78	1.29e-1	139.18	5.95e-2	40.92	3.96e-3	12.03	**3.26e-3**	**9.95**
80%	5.99e-1	238.06	9.19e-2	80.37	2.90e-1	135.12	1.26e-1	106.91	9.10e-3	17.48	**6.27e-3**	**13.79**
20 × 20 × 20 × 20 × 20												
30%	4.63e-1	66.18	1.22e-2	60.79	4.27e-2	161.67	2.89e-3	18.63	7.91e-4	**3.70**	**3.07e-4**	4.92
50%	6.00e-1	58.37	5.98e-2	68.96	8.18e-2	160.00	6.58e-3	29.25	7.15e-4	**6.96**	**6.00e-4**	8.92
70%	7.06e-1	50.39	8.59e-2	80.89	1.68e-1	174.45	9.58e-2	65.25	1.40e-3	18.37	**1.20e-3**	**13.28**
80%	7.40e-1	96.97	1.05e-1	94.42	1.78e-1	175.31	8.18e-2	101.26	5.91e-2	23.08	**1.40e-2**	**20.32**

Bold values indicate the best results obtained from the experiments.

**Figure 3:**
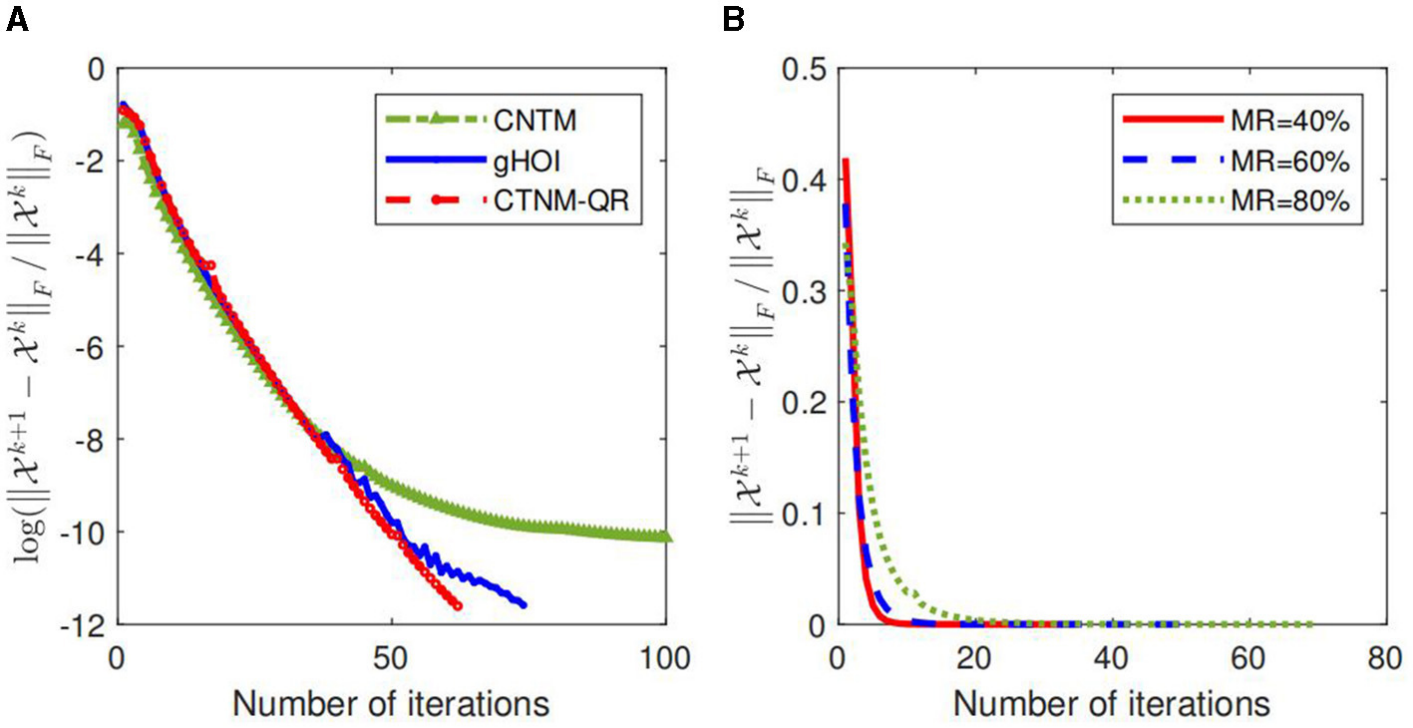
Convergence of the algorithm on synthetic data with the size 100 × 100 × 100. **(A)** Log-value of relative change of the CTNM, gHOI, and CTNM-QR with 80% MR. **(B)** Relative changes of the CTNM-QR with various MRs.

We also investigate the convergence of the CTNM, gHOI, and CTNM-QR methods on synthetic tensor data with the Tucker-rank (*R*_1_, *R*_2_, *R*_3_) = (10, 10, 10) and the size 100 × 100 × 100, as shown in Figure 3A, where the abscissa indicates the number of iterations and the vertical indicates the log-value of the relative change of the X^*k*^. The results display that the relative change of the convergence of the CTNM-QR drops more quickly and smoothly than that of CTNM and gHOI. Figure 3B illustrates the relative changes of the convergence of the CTNM-QR approach at 40, 60, and 80% MRs. From the Figure 3B, we find that even at the high MR, the CTNM-QR converges fast in fewer than 100 iterations.

### 4.2 Color image completion

**Figure 4:**
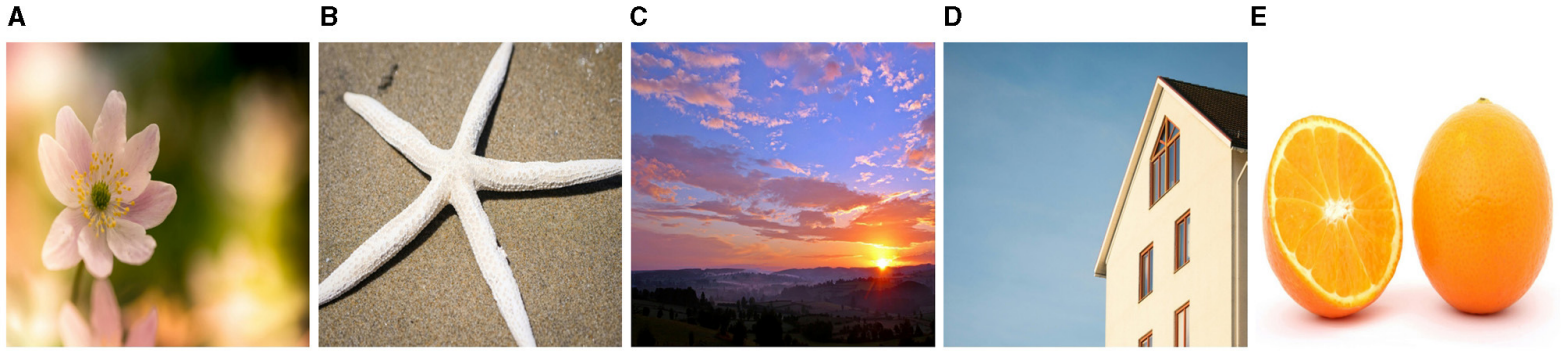
The original color images. **(A)** Flower (taken from https://www.pexels.com/Nathalie De Boever), **(B)** Starfish (taken from https://www.pexels.com/Kindel Media), **(C)** Sky (taken from https://www.pexels.com/Pixabay), **(D)** House (taken from https://www.pexels.com/Pixabay), **(E)** Orange (taken from https://www.pexels.com/Pixabay).

In this subsection, we confirm the efficacy of the CTNM-QR by performing recovery on RGB real-color images, where each image is represented as a third-order tensor. The low-rank nature of color images has been demonstrated in Figure 1, which allows excellent recovery of incomplete images utilizing the low-rank structure of the tensor. The low-rank color pictures chosen for this experiment include Flower, Starfish, Sky, Lena, and Apple. The original images are portrayed in Figure 4. The images are 500 × 500 × 3 tensors and encompass portraits and various natural landscapes. The image pixels are normalized to [0, 1]. We carry out multiple experimental comparisons for every image in order to determine the optimal rank value.

In the concrete implementation, the tolerance error of all algorithms is set to *tol* = 10^−4^, the maximum number of iterations *K* = 5 × 10^5^ for TT-SGD and *K* = 500 for the rest of the algorithms. For the FaLRTC, gHOI, CTNM and CTNM-QR, we set the weights $\alpha_{i}=\left[1,1,10^{-3}\right]$, and the smoothing parameter for the former to $\alpha_{i}/\sqrt{I_{i}}$ for *i* = 1, 2, 3. For the CTNM-QR, we set $\mu_{0}=10^{-3}$, $\mu_{\max}=10^{10}$, ρ = 1.05. The rest of the algorithm parameters are preserved at the default settings.

For the color images, we utilize random uniform sampling to make them missing, and the average reconstruction results from 100 independent experiments, including RSE and time cost (seconds), are reported in Table 3, where MR is set to 40, 60, and 80%. The best results are highlighted in bold in the table, and it is clear that the proposed CTNM-QR method achieves the best RSE values in all images and outperforms the other competing methods in terms of CPU time in a substantial number of cases. For example, when recovering Sky with an MR of 80%, the RSE of the CTNM-QR is improved by 0.036 and 0.071 over gHOI and the original algorithm CTNM, respectively, and by 0.119 over the FaLRTC. In terms of running speed, it outperforms the CTNM and the TT-SGD by around 4 and 7 times, respectively. Furthermore, in the majority of image completions, the CP-WOPT method takes the longest. It is obvious that the CTNM-QR can efficiently mine the low-rank image spatial correlation to precisely recover the missing tensor in a reduced iteration time.

**Table 3:** RSE and time cost (seconds) comparison on true color images at different MRs.

**MR**	**Method**	**Flower**		**Starfish**		**Sky**		**Lena**		**Apple**	
		**RSE**	**Time**	**RSE**	**Time**	**RSE**	**Time**	**RSE**	**Time**	**RSE**	**Time**
40%	CP-WOPT	7.46e-2	210.25	1.88e-1	186.98	6.58e-2	40.26	1.79e-1	68.70	6.93e-2	100.02
	FaLRTC	1.26e-1	38.50	1.74e-1	44.21	4.47e-2	34.69	1.19e-1	38.79	6.69e-2	48.69
	TT-SGD	1.12e-1	75.05	1.76e-1	77.73	5.56e-2	61.59	1.11e-1	61.10	6.90e-2	69.15
	gHOI	5.32e-2	17.91	1.20e-1	**12.83**	3.24e-2	9.78	9.59e-2	13.48	3.18e-2	9.43
	CTNM	8.24e-2	19.35	1.69e-1	34.58	2.95e-2	8.60	1.13e-1	26.39	3.66e-2	11.27
	CTNM-QR	**4.16e-2**	**11.31**	**1.15e-1**	14.54	**2.56e-2**	**4.19**	**8.88e-2**	**7.84**	**3.02e-2**	**3.29**
60%	CP-WOPT	1.22e-1	204.18	1.97e-1	350.43	9.03e-2	52.39	1.96e-1	159.59	1.16e-1	115.27
	FaLRTC	1.93e-1	37.74	2.51e-1	53.79	6.50e-2	43.14	1.76e-1	45.32	9.14e-2	66.90
	TT-SGD	1.35e-1	69.01	2.19e-1	79.79	6.55e-2	64.15	1.37e-1	74.22	8.54e-2	79.98
	gHOI	7.97e-2	20.46	1.64e-1	**15.34**	3.68e-2	10.52	1.11e-1	16.48	4.24e-2	12.37
	CTNM	1.45e-1	35.46	2.79e-1	45.18	3.51e-2	14.60	1.54e-1	37.47	4.05e-2	14.02
	CTNM-QR	**5.67e-2**	**18.26**	**1.43e-1**	17.79	**3.08e-2**	**8.02**	**1.02e-1**	**14.39**	**3.78e-2**	**7.30**
80%	CP-WOPT	1.91e-1	185.96	2.83e-1	181.91	1.37e-1	61.59	2.41e-1	80.00	1.86e-1	130.56
	FaLRTC	3.06e-1	48.92	3.71e-1	59.68	1.58e-1	48.67	2.70e-1	57.96	1.06e-1	57.57
	TT-SGD	1.57e-1	77.56	2.66e-1	69.92	9.20e-2	68.32	1.67e-1	71.20	9.41e-2	73.87
	gHOI	1.41e-1	**27.82**	2.12e-1	22.37	7.53e-2	14.53	1.34e-1	**18.25**	7.45e-2	21.52
	CTNM	1.99e-1	50.89	3.36e-1	50.87	1.10e-1	47.72	1.88e-1	49.71	7.78e-2	28.82
	CTNM-QR	**9.13e-2**	30.45	**1.79e-1**	**19.91**	**3.90e-2**	**12.64**	**1.10e-1**	20.58	**4.59e-2**	**15.12**

Bold values indicate the best results obtained from the experiments.

**Figure 5:**
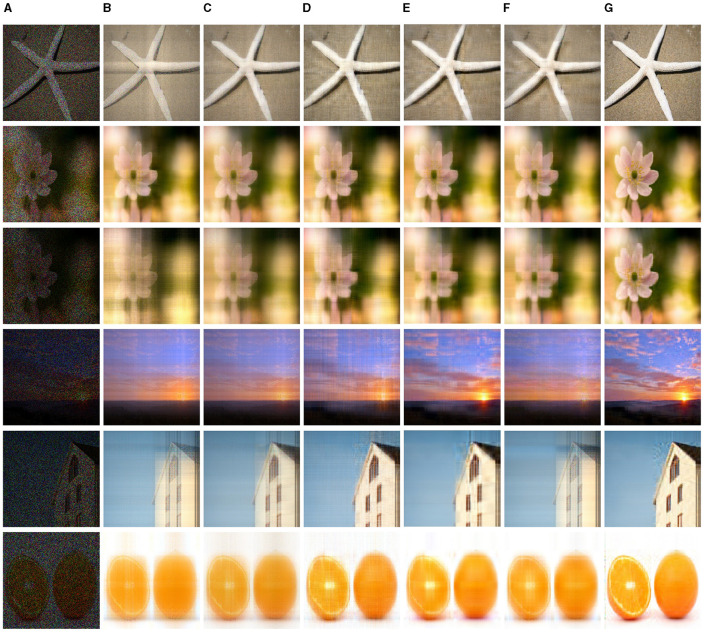
Illustration of image completion performance comparisons. The first two rows display the results for a missing rate MR = 60%, and the last four rows display the results for a missing rate MR = 80% (best viewed zoomed in). **(A)** Observation. **(B)** CP-WOPT. **(C)** FaLRTC. **(D)** TT-SGD. **(E)** gHOI. **(F)** CTNM. **(G)** CTNM-QR. Adapted from https://pic.sogou.com.

Figure 5 depicts visual comparisons of the observed images with the images following the various completion algorithms, where the missing rate of incomplete images is 60 and 80%, and the missing entries are displayed in black. From Figure 5, it can be seen that the best visibility outcomes are obtained by the CTNM-QR method (Figure 5G), and the recovered image is very clear and close to the original image. In most instances, the results of the CP-WOPT contain some fairly ambiguous parts, and this is the most acute, especially for the Lena and Apple. In the completion of missing data for images with an 80% MR, the FaLRTC and CTNM exhibit suboptimal performance, particularly on the Flower and Lena datasets. Although the results of the gHOI and TT-SGD appear to be more discernible in comparison, the finer details of the images have not been fully recovered, indicating a certain degree of information loss. It implies that the proposed CTNM-QR method can recover the images accurately and efficiently.

### 4.3 Brain MRI completion

In the subsection, we evaluate the performance of the CTNM-QR algorithm via the MRI images completion of the brain. MRI images of the brain are acquired through the use of magnetic resonance imaging, which utilizes a strong magnetic field and harmless radio waves. It has an influential role in the fields of medicine and neuroscience, being a key tool used by physicians to diagnose diseases, study brain activity, and guide treatment programs. Here, we analyze brain MRI data with the size 236 × 236 × 180. Similarly, medical MRI data is approximated as a low-rank tensor and thus can be recovered with low-rank completion algorithms. For comparison, the tolerance value for all algorithms is set to *tol* = 10^−3^, the tensor Tucker-rank is set to *R*_1_ = *R*_2_ = *R*_3_ = 30 for the gHOI, CTNM, and CTNM-QR, the tensor CP rank is set to 40 for the CP-WOPT, and the TT rank is set to *R*_1_ = *R*_2_ = 20 for the TT-SGD. We choose a slice of the MRI images for completion due to their enormous computation cost.

**Figure 6:**
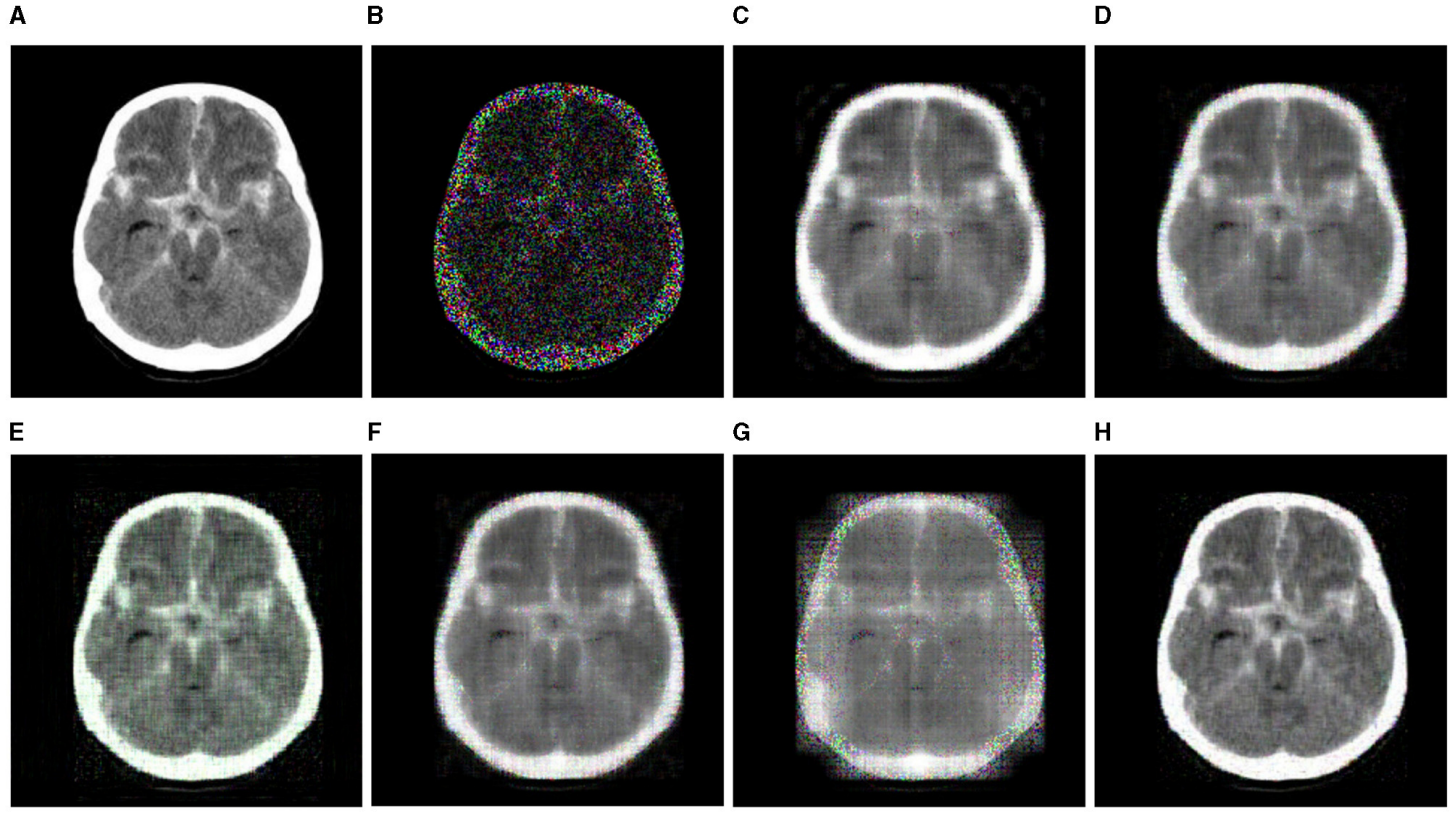
Completion visualization results of brain MRI slice data at 70% MR (best viewed zoomed in). **(A)** Original (adapted from https://www.kaggle.com/datasets/tourist55/alzheimers-dataset-4-class-of-images). **(B)** Observation. **(C)** CP-WOPT. **(D)** FaLRTC. **(E)** TT-SGD. **(F)** gHOI. **(G)** CTNM. **(H)** CTNM-QR.

Table 4 presents the recovery accuracy (RSE) and time cost (seconds) of every algorithm at varying MRs, where the MRs are set to 30, 50, and 70%, respectively. The findings show that the RSE of the CTNM-QR, gHOI, and CTNM are nearly equal at MR values of 30 and 50%. However, when the MR increases to 70%, the RSE of the CTNM and gHOI dramatically rose. In terms of the runtime, the proposed CTNM-QR method typically runs faster than others, especially more noticeable at the higher MR. In addition, Figure 6 displays the recovery results of the brain MRI slice data at the 70% MR, where the CTNM-QR outperforms the other algorithms in terms of both recovered vision and efficiency.

**Table 4:** RSE and time cost (seconds) comparison on brain MRI scanning slice.

	**CP-WOPT**		**FaLRTC**		**TT-SGD**		**CTNM**		**gHOI**		**CTNM-QR**	
**MR**	**RSE**	**Time**	**RSE**	**Time**	**RSE**	**Time**	**RSE**	**Time**	**RSE**	**Time**	**RSE**	**Time**
30%	9.63e-2	13.69	7.25e-2	8.29	9.75e-2	74.36	4.90e-2	4.59	4.90e-2	4.10	**4.89e-2**	**3.34**
50%	1.19e-1	16.56	1.11e-1	9.98	1.29e-1	73.36	6.83e-2	7.07	6.84e-2	8.86	**6.80e-2**	**5.60**
70%	1.89e-1	25.27	1.70e-1	13.30	1.57e-1	69.38	3.79e-1	22.92	1.97e-1	11.59	**1.21e-1**	**7.16**

Bold values indicate the best results obtained from the experiments.

## 5 Conclusion

In this paper, we propose a CTNM-QR method, which is based on an enhancement of CTNM and aims at addressing the problem of completing the low-rank missing tensor. Firstly, we review the CTNM algorithm briefly and discuss our changes, which can be categorized into two major points. One difference is that the model's auxiliary variables are introduced as tensors rather than matrix versions, and the tensor form is able to utilize multidimensional information to better retain the spatial structure of high-dimensional data. The other is that the solution of the factor matrix in the model flexibly adopts the thin QR decomposition instead of HOOI, which avoids the computation of multiple SVDs in each loop and minimizes the computational cost to a certain extent, thus speeding up the process. Secondly, we further investigate the convergence and complexity of the CTNM-QR method. Ultimately, extensive experiments on synthetic data of various orders, real color images, and brain MRI data indicate that our method not only beats most state-of-the-art LRTC algorithms in terms of completion accuracy and visualization, but also runs faster.

In the future, we will continue to explore how to automatically determine the optimal rank of a tensor in CTNM-QR to settle the rank selection challenge. In addition, combining sophisticated techniques such as deep learning will boost the algorithm's ability to adapt to more complicated data. To widen the method's application scenarios, we are interested in adapting CTNM-QR to additional practical applications such as recommender systems, video denoising, and so on.

## Data availability statement

The original contributions presented in the study are included in the article/supplementary material, further inquiries can be directed to the corresponding author.

## Author contributions

YW: Writing – original draft. YJ: Writing – review & editing.

## References

[B1] Acar E. Dunlavy D. M. Kolda T. G. Mørup M. (2011). Scalable tensor factorizations for incomplete data. Chemomet. Intell. Lab. Syst. 106, 41–56. 10.1016/j.chemolab.2010.08.00431251750

[B2] Bai Z. Li Y. Woźniak M. Zhou M. Li D. (2021). Decomvqanet: decomposing visual question answering deep network via tensor decomposition and regression. Pattern Recognit. 110:107538. 10.1016/j.patcog.2020.107538

[B3] Bengua J. A. Phien H. N. Tuan H. D. Do M. N. (2017). Efficient tensor completion for color image and video recovery: low-rank tensor train. IEEE Transact. Image Process. 26, 2466–2479. 10.1109/TIP.2017.2672439 28237929

[B4] Boţ R. I. Nguyen D.-K. (2020). The proximal alternating direction method of multipliers in the nonconvex setting: convergence analysis and rates. Math. Operat. Res. 45, 682–712. 10.1287/moor.2019.100819642375

[B5] Boumal N. (2023). An Introduction to Optimization on Smooth Manifolds. Cambridge: Cambridge University Press.

[B6] Cai J.-F. Candès E. J. Shen Z. (2010). A singular value thresholding algorithm for matrix completion. SIAM J. Optimiz. 20, 1956–1982. 10.1137/080738970

[B7] Candes E. Recht B. (2012). Exact matrix completion via convex optimization. Commun. ACM 55, 111–119. 10.1145/2184319.2184343

[B8] Cattell R. B. (1944). Parallel proportional profiles and other principles for determining the choice of factors by rotation. Psychometrika 9, 267–283. 10.1007/BF02288739

[B9] Chao Z. Huang L. Needell D. (2021). Hosvd-based algorithm for weighted tensor completion. J. Imaging 7:110. 10.3390/jimaging7070110 PMC8321375 39080898

[B10] De Lathauwer L. De Moor B. Vandewalle J. (2000a). A multilinear singular value decomposition. SIAM J. Matrix Anal. Appl. 21, 1253–1278. 10.1137/S0895479896305696

[B11] De Lathauwer L. De Moor B. Vandewalle J. (2000b). On the best rank-1 and rank-(r1, r2,..., rn) approximation of higher-order tensors. SIAM J. Matrix Anal. Appl. 21, 1324–1342. 10.1137/S0895479898346995

[B12] Filipović M. Jukić A. (2015). Tucker factorization with missing data with application to low-n-rank tensor completion. Multidimens. Syst. Signal Process. 26, 677–692. 10.1007/s11045-013-0269-924457512

[B13] Gandy S. Recht B. Yamada I. (2011). Tensor completion and low-n-rank tensor recovery via convex optimization. Inverse Probl. 27:025010. 10.1088/0266-5611/27/2/025010

[B14] Glowinski R. (2014). On Alternating Direction Methods of Multipliers: A Historical Perspective. Modeling, Simulation and Optimization for Science and Technology. Berlin: Springer, 59–82.

[B15] Han D.-R. (2022). A survey on some recent developments of alternating direction method of multipliers. J. Operat. Res. Soc. China 10, 1–52. 10.1007/s40305-021-00368-336141170

[B16] Hillar C. J. Lim L.-H. (2013). Most tensor problems are np-hard. J. ACM 60, 1–39. 10.1145/2512329

[B17] Ji L. Musialski P. Wonka P. Jieping Y. (2009). Tensor completion for estimating missing values in visual data, in International Conference on Computer Vision (Kyoto: IEEE), 2114–2121.

[B18] Kajo I. Kamel N. Ruichek Y. (2019). Incremental tensor-based completion method for detection of stationary foreground objects. IEEE Transact. Circ. Syst. Video Technol. 29, 1325–1338. 10.1109/TCSVT.2018.2841825

[B19] Kasai H. Mishra B. (2016). Low-rank tensor completion: a riemannian manifold preconditioning approach, in Proceedings of the 33rd International Conference on Machine Learning, Vol. 48 (New York, NY: PMLR), 1012–1021.

[B20] Kolda T. G. Bader B. W. (2009). Tensor decompositions and applications. SIAM Rev. 51, 455–500. 10.1137/07070111X

[B21] Kressner D. Steinlechner M. Vandereycken B. (2014). Low-rank tensor completion by riemannian optimization. BIT Numer. Math. 54, 447–468. 10.1007/s10543-013-0455-z

[B22] Liu J. Musialski P. Wonka P. Ye J. (2013). Tensor completion for estimating missing values in visual data. IEEE Trans. Pattern Anal. Mach. Intell. 35, 208–220. 10.1109/TPAMI.2012.39 22271823

[B23] Liu Y. (2013). Algorithm Research of Fast Low-Rank Matrix and Tensor Recovery (PhD thesis). Wanfang Data Resource System.

[B24] Liu Y. Long Z. Huang H. Zhu C. (2019a). Low cp rank and tucker rank tensor completion for estimating missing components in image data. IEEE Transact. Circ. Syst. Video Technol. 30, 944–954. 10.1109/TCSVT.2019.2901311

[B25] Liu Y. Long Z. Zhu C. (2019b). Image completion using low tensor tree rank and total variation minimization. IEEE Transact. Multim. 21, 338–350. 10.1109/TMM.2018.2859026

[B26] Liu Y. Shang F. Cheng H. Cheng J. Tong H. (2014a). Factor matrix trace norm minimization for low-rank tensor completion, in Proceedings of the 2014 SIAM International Conference on Data Mining (SDM) (Philadelphia, PA: Society for Industrial and Applied Mathematics (SIAM)), 866–874.

[B27] Liu Y. Shang F. Fan W. Cheng J. Cheng H. (2014b). Generalized higher-order orthogonal iteration for tensor decomposition and completion. Adv. Neural Inf. Process. Syst. 27, 1–9. 10.5555/2968826.2969023 26595932

[B28] Miao J. Kou K. I. Liu W. (2020). Low-rank quaternion tensor completion for recovering color videos and images. Pattern Recognit. 107:107505. 10.1016/j.patcog.2020.107505

[B29] Mu C. Huang B. Wright J. Goldfarb D. (2014). Square deal: lower bounds and improved relaxations for tensor recovery, in Proceedings of the 31st International Conference on Machine Learning, Vol. 32 (Beijing: PMLR), 73–81.

[B30] Oseledets I. V. (2011). Tensor-train decomposition. SIAM J. Sci. Comp. 33, 2295–2317. 10.1137/090752286

[B31] Panagakis Y. Kossaifi J. Chrysos G. G. Oldfield J. Nicolaou M. A. Anandkumar A.. (2021). Tensor methods in computer vision and deep learning. Proc. IEEE 109, 863–890. 10.1109/JPROC.2021.3074329

[B32] Qiu Y. Zhou G. Zhao Q. Xie S. (2022). Noisy tensor completion via low-rank tensor ring. IEEE Transact. Neural Netw. Learn. Syst. 35, 1127–1141. 10.1109/TNNLS.2022.3181378 35714084

[B33] Romera-Paredes B. Pontil M. (2013). A new convex relaxation for tensor completion, in Proceedings of the 26th International Conference on Neural Information Processing Systems, Vol. 2 (New York, NY: Curran Associates Inc.), 2967–2975.

[B34] Shang F. Liu Y. Cheng J. Yan D. (2017). Fuzzy double trace norm minimization for recommendation systems. IEEE Transact. Fuzzy Syst. 26, 2039–2049. 10.1109/TFUZZ.2017.2760287

[B35] Shi J. Yang W. Yong L. Zheng X. (2015). Low-rank tensor completion via tucker decompositions. J. Comp. Inf. Syst. 11, 3759–3768. 10.12733/jcis14329

[B36] Shi Q. Lu H. Cheung Y.-m. (2017). Tensor rank estimation and completion via cp-based nuclear norm, in Proceedings of the 2017 ACM on Conference on Information and Knowledge Management (CIKM) (New York, NY: Association for Computing Machinery), 949–958.

[B37] Signoretto M. De Lathauwer L. Suykens J. A. (2010). Nuclear Norms for Tensors and Their Use for Convex Multilinear Estimation. Submitted to Linear Algebra and Its Applications. Amsterdam: Elsevier, 43.

[B38] Su L. Liu J. Tian X. Huang K. Tan S. (2022). Iterative tensor eigen rank minimization for low-rank tensor completion. Inf. Sci. 616, 303–329. 10.1016/j.ins.2022.10.061

[B39] Tucker L. R. (1963). Implications of factor analysis of three-way matrices for measurement of change. Probl. Meas. Change 15, 122–137. 10.1108/09534810210423008

[B40] Xu K. Zhang Y. Xiong Z. (2021). Iterative rank-one matrix completion via singular value decomposition and nuclear norm regularization. Inf. Sci. 578, 574–591. 10.1016/j.ins.2021.07.035

[B41] Yoshii K. Kitamura K. Bando Y. Nakamura E. Kawahara T. (2018). Independent low-rank tensor analysis for audio source separation, in 2018 26th European Signal Processing Conference (EUSIPCO) (IEEE), 1657–1661.

[B42] Yu Q. Zhang X. Chen Y. Qi L. (2023). Low tucker rank tensor completion using a symmetric block coordinate descent method. Numer. Linear Algebra Appl. 30:e2464. 10.1002/nla.2464

[B43] Yuan L. Li C. Mandic D. Cao J. Zhao Q. (2019a). Tensor ring decomposition with rank minimization on latent space: an efficient approach for tensor completion, in Proceedings of the AAAI Conference on Artificial Intelligence, Vol. 33 (New York, NY: AAAI Press), 9151–9158.

[B44] Yuan L. Zhao Q. Cao J. (2017). Completion of high order tensor data with missing entries via tensor-train decomposition, in International Conference on Neural Information Processing (Berlin: Springer), 222–229.

[B45] Yuan L. Zhao Q. Gui L. Cao J. (2019b). High-order tensor completion via gradient-based optimization under tensor train format. Signal Process. Image Commun. 73, 53–61. 10.1016/j.image.2018.11.012

[B46] Zhang Z. Aeron S. (2016). Exact tensor completion using t-svd. IEEE Transact. Signal Process. 65, 1511–1526. 10.1109/TSP.2016.263946635259105

[B47] Zhao X.-L. Xu W.-H. Jiang T.-X. Wang Y. Ng M. K. (2020). Deep plug-and-play prior for low-rank tensor completion. Neurocomputing 400, 137–149. 10.1016/j.neucom.2020.03.018

[B48] Zhou P. Lu C. Lin Z. Zhang C. (2018). Tensor factorization for low-rank tensor completion. IEEE Transact. Image Process. 27, 1152–1163. 10.1109/TIP.2017.2762595 29028199

